# Shared-weight graph framework for comprehensive protein stability prediction across diverse mutation types

**DOI:** 10.1093/bib/bbaf190

**Published:** 2025-04-24

**Authors:** Gen Li, Sijie Yao, Long Fan

**Affiliations:** Production and R&D Center I of LSS, GenScript (Shanghai) Biotech Co., Ltd., 186 He Dan Road, Pudong New Area, Shanghai 200131, China; Production and R&D Center I of LSS, GenScript (Shanghai) Biotech Co., Ltd., 186 He Dan Road, Pudong New Area, Shanghai 200131, China; Production and R&D Center I of LSS, GenScript (Shanghai) Biotech Co., Ltd., 186 He Dan Road, Pudong New Area, Shanghai 200131, China

**Keywords:** protein stability, deep learning, shared-weight, diverse mutation, protein embedding

## Abstract

Research on protein stability changes is vital for understanding disease mechanisms and optimizing industrial enzymes. Protein thermal stability can be modified by variants leading to changes in ΔΔG values between wild-type and mutant proteins. Despite advances, most models focus on single-point mutations, overlooking multipoint and indel mutations. Typically, the single-point mutation is expected to have a relatively limited impact on the function of a protein, necessitating more drastic modifications to meet new challenges. Current methods for multipoint mutations yield poor results, and no method exists for any length of indel mutations. To address this, we introduce UniMutStab, a shared-graph convolutional network leveraging protein language models and residue interaction networks to access any type of mutation. An embedded edge weight module enhances the integration of residue node features and interactions, improving prediction accuracy. Trained on the “Mega-scale” dataset with ~780 000 mutations, UniMutStab surpasses existing methods in predicting protein stability changes. It is a purely sequence-based approach to predict arbitrary mutation types, demonstrating robust generalization across multiple tasks and potentially contributing significantly to protein engineering, personalized therapeutics, and diagnostic methodologies.

## Introduction

Proteins constitute a remarkably versatile and functional group of biomolecules that have been harnessed for various applications in medical, industrial, and scientific domains [1, 2]. Their thermal stability refers to the ability of proteins to maintain their 3D structure in environments, which is fundamental for the functional activity of proteins [3]. Protein variants can alter protein folding conformation, leading to differences in thermal stability between wild-type and mutant proteins, which are reflected in changes in ΔΔG values, the difference in Gibbs free energy change (ΔG) between the wild-type protein and its mutant form [4, 5]. While stability changes can be determined using experimental methods [6, 7], computational methods offer the potential for competitive, rapid, and easily accessible research frameworks. To assess the ΔΔG of protein, numerous prediction methods, such as force field [8], empirical or knowledge-based [9], machine learning [10], and deep learning [11], have been used for protein stability prediction. In recent years, with continuous updates in data, hardware, and methodologies, machine learning and even deep learning approaches have made some breakthroughs in predicting ΔΔG, gradually becoming the mainstream method for predicting changes in protein mutation thermal stability.

Typically, the single-point mutation is expected to have a relatively limited impact on the function of proteins, necessitating more extensive modifications to meet new challenges [12]. However, almost all existing prediction models focus on the stability changes of single-point [13, 14], insertion/deletion (indel), and even multipoint mutations were ignored. So far, only a few methods can evaluate the multipoint with poor results [15–17], as for the indels, although some methods, such as PROVEAN [18] and INDELi [19] (the code does not support multiple indels), can assess the effect of indel variation. They are only applicable to evaluate the effects of variants that insert or delete a single amino acid. In other words, the ability to truly assess the stability changes of any mutation is still missing. Data and methods are two potential barriers that may prevent people from developing a novel method for predicting protein stability changes upon indel mutations, the development of a faster and more accurate model is imperative for the design of bespoke proteins applicable to the fields of protein engineering, personalized therapeutics, and precise diagnostic methodologies [4].

Many models such as UniRep [20], TAPE [21], ProtTrans [22], and Evolutionary Scale Modeling (EMS) [23] demonstrate the power of deep learning, especially natural language processing, in zero-shot prediction and semantic representation of proteins. Based on this insightful observation, we introduce UniMutStab, an innovative shared-graph convolutional network carefully designed to exploit the synergistic effect of protein language models and residue interaction networks and provide a method for evaluating indels. An edge weight module is embedded in the UniMutStab framework, coordinating the seamless integration between residue node features and residue interactions. This integration significantly improves the accuracy of predicting stability changes of multiple point mutations. In addition, to enhance the feature representation capability of our method, we introduce a global protein-embedding layer and residue interaction relationships. These features help capture a wide range of relevant information related to protein function and structure, thereby enriching the model’s predictive power in the field of protein stability.

To help solve the data shortage of different mutation types, UniMutStab was trained on the newly published “Mega-scale” dataset [24], which is a massive, universal, and diverse dataset that includes ~780 000 with single, multiple, and indel mutations. Moreover, it surpasses current leading-edge methods in dealing with various mutation-induced protein stability issues, as evidenced by its performance across multiple independent datasets used for benchmarking. The accurate prediction of *de novo* proteins and the correct classification of temperature adaptability of bacterial proteomes demonstrate the robust generalization ability of our method. Collectively, UniMutStab stands as a significant contribution to the field, showcasing a comprehensive approach toward advancing predictive models for protein engineering, evolutionary analysis, and disease diagnosis.

## Method

### Dataset preparation

#### Training set

Tsuboyama *et al*. [24] developed complementary DNA (cDNA) display proteolysis for the high-through measurement of thermodynamic folding stability for up to 900 000 sequences in 1 week. This dataset contains single-point data, as well as multipoint and indel data. The raw data need to remove some unreasonable points to enhance the robustness of the model. The removed data satisfied the following rules: (i) points marked as unreliable in Tsuboyama’s work and (ii) data with unnatural proteins. In the original dataset, the number of stabilizing mutations is less than the number of destabilizing mutations. To balance the stabilizing data and destabilizing data, we reversed and merged all the data to satisfy the antisymmetric property (direct: wildperty (=ildperty (diremutantrty (= -tant [20–22]. This yielded a final training set of 651 816 mutations across 230 proteins.

#### Blind test set

We selected several datasets from other published papers to test the performance of different methods. S669 is a widely used single-point mutation dataset that is easy to compare with other methods [14]. S96, another single-point mutation dataset, is used for additional independent testing. M28 is a dataset of multiple point variants created by Montanucci *et al*. [25]. M38 is a new dataset of multiple point variants derived from the four databases: MPTherm [16], ProthermDB [17], ThermoMutDB [18], and FireProtDB [19]. S98, a “variant-to-variant” single-point mutation dataset, is the data of selecting sequences that already contain mutations in the M38 dataset and mutating them again. In other words, some papers continue to induce mutations after initial sequence variations to further enhance the thermal stability of the protein. M218 does the same as S98 except the mutation point is multiple. We additionally designed three Indel blind test sets to test the performance differences of currently available methods. It consists of three proteins with experimental ΔΔG from the papers [4, 26, 27]. Indels732 is a test set of 732 indel samples selected from cDNA data, and all protein sequences are artificially synthesized sequences. Notably, except for the sets isolated from cDNA data, all the blind test sets comply with the rule of <25% similarity with the training set.

Table S1 summarizes the datasets used in this study. For some datasets obtained from other works, we changed the name of the dataset because we removed some samples that did not meet the sequence identity requirement with the training set.

### The optimum growth temperature dataset

We downloaded an OGT (optimum growth temperature) dataset from the TEMPURA database [28] (http://togodb.org/db/tempura) on 21 March 2024. This dataset includes 8639 prokaryotic species (comprising both bacteria and archaea) and >15 000 proteins, optimum growth temperature ranges from <5°C for psychrophilic bacteria to >60°C for thermophilic bacteria. For data cleaning and preprocessing, we utilized a Python script to load the CSV file, check for consistency and completeness, and remove missing and outlier values. All protein sequences were obtained by searching the species ID in the Uniprot database. We standardized the data format to ensure that each record contained the species name, optimal growth temperature, and the corresponding protein sequences. Finally, the OGT dataset includes 14 987 samples from 8621 prokaryotic species.

### Disease-related mutations dataset

The data of pathogenic and benign amino acid variants were downloaded from the ClinVar2 database on 25 March 2024 by customized Python scripts. The dataset includes the UniProt ID, sequence, mutation information, and effect. Since samples may use different synonyms to describe whether a mutation is pathogenic or benign, we defined all labels containing “pathogenic” as positive and those containing “benign” as negative. The filtering rules are as follows: (i) nonredundant data. (ii) Only keep results with clear meanings. (iii) Remove those protein sequences containing non-standard amino acids. (iv) Remove sequences longer than 1 K. To ensure a balance between positive and negative samples, we arbitrarily selected the same number of benign mutations as pathogenic data but not for indels because there were fewer negative data. After that, positive samples and negative samples are the same including 21 192 single mutations, respectively. There were 1086 and 422 indels mutations for positive and negative samples, respectively.

### Training procedure

#### Graph neural network architecture

We used the SageConv architecture as the model framework to train the UniMutStab, a popular variant GNN (graph neural network) approach that learns node embeddings by gathering information from nearby nodes. SageConv improves the GNN, making convolution operations more expressive and able to capture more complex features. It includes two advantages: normalized aggregation functions are used to capture subtle differences, and skip connections are used to mitigate gradient vanishing and boost matrix operations [29]. In our model, the wild type and mutant are represented by separate graphs, and parameters are then shared in the graph neural network. Both wild and mutant graphs are used to process variable-length sequences. Each network layer takes the graph from the previous layer and outputs it to the next layer. The pooling layer, fully connected layer, and last layer follow this, which is used to output the predicted ΔΔG. We built the model using the DGL Pytorch framework [30], and the pseudocode to describe the UniMutStab workflows can be found in Supplementary Information.

#### Node features

Node features often use one-hot algorithms to encode the physicochemical or evolutionary information of residues. In this work, we use the protein language model to capture whole protein features. Prediction methods using ProtT5-XL-Uniref50 [22] have been shown to have superior performance in several downstream tasks, such as solubility, contact map, and function prediction. We obtained an N^*^1024 matrix through the last layer of the ProtT5-XL-Uniref50 pretrained model, where *N* is the length of the sequence and 1024 is the size of the hidden layer. We input the wild-type sequence and the mutant sequence, respectively, to obtain the node features represented as the entire sequence.

#### Adjacency matrix

In a graph, the adjacency matrix consists of either 0 or 1, where 1 means a connection exists and 0 does not. The protein structure can be regarded as a graph in which each amino acid is directly connected according to a certain relationship. The protein network can represent the adjacency matrix through the distance of the residues. If the distance between the nodes is less than a certain distance, the nodes are considered to be directly connected. Then the embedding corresponding to each residue is assigned to the node on the protein graph. We selected the SPOT-Contact-LM [31] to predict the residue contact map as the input of the graph, where the cutoff is set to 0.1.

#### Edge weight

For unweighted graphs, entries in the adjacency matrix can be either 0 or 1, where a single-valued entry represents an edge. In a weighted graph, the values associated with each connection, represented by the edges, can be any meaningful numerical figure. These weights often carry significant context, such as representing the strength of a connection or a confidence score. In our work, the edge weight used the probability value output by SPOT-Contact-LM [31].

#### Model hyperparameter

We used the grid-search technique to determine the best parameter combination based on performance on five cross-validation results. To avoid overfitting, we use an early stopping criterion with patience 5. In addition, an Adam optimizer optimizes the model. The Pearson correlation coefficient and the root-mean-squared error were used to monitor the training loss within 20 epochs. The detailed parameters for each model can be found in the JSON file.

## Result

### UniMutStab used sequence embeddings in a shared graph network

The prediction of protein stability change is a regression task; thus, we propose a novel SageConv method named UniMutStab. SageConv is a variant of GNNs successfully applied to multiple tasks, including node classification, link prediction, and graph classification. In classification tasks, it has been used to classify protein–ligand [32] and protein function [33] interaction networks. A graph is the basic component of SageConv, which consists of nodes (vertices) and edges. In graph theory, graph *G* = (*V*, *E*, *W*) typically represents a weighted graph where: *V* and *E* represent the set of nodes and edges, respectively. In addition, for each edge in *E*, there is an associated weight in *W*. These weights can represent distances, scores, or any other values that might be associated with the edges of the graph.

**Figure 1:**
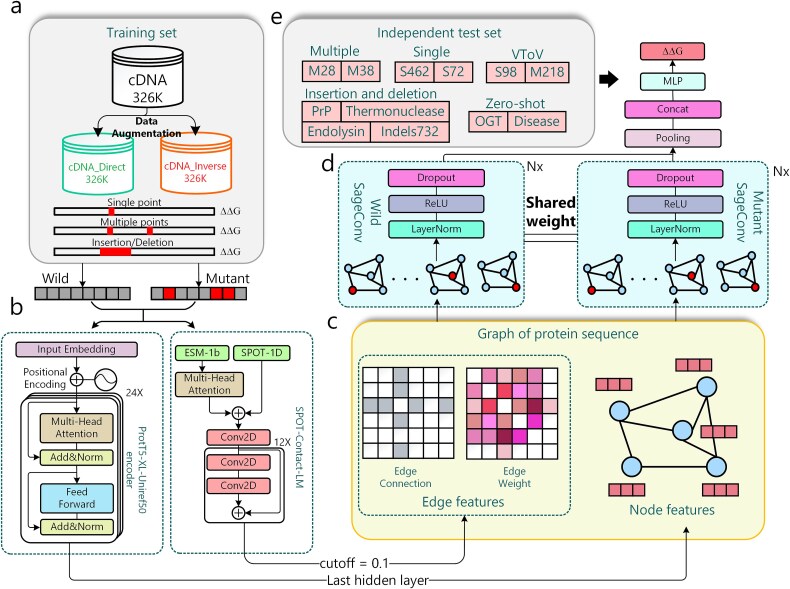
Schematic diagram of the development and testing for UniMutStab. (a) The training set was taken from K50_dG_Dataset1_Dataset2.Csv and merged with the inversed data. The final set includes 651 816 single/multiple/indel mutations across 230 proteins. (b) The graph node features are created by the last hidden layer of the ProtT5-XL-Uniref50 pretrained model. The edge connectivity information and the corresponding weights are extracted from the SPOT-contact-LM model. The dashed box indicates the existing method we use as a feature extractor. (c) The protein sequence graph is composed of features extracted by existing models. (d) The major architecture of UniMutStab is composed of a wild-type protein sequence graph and a mutant protein sequence graph, and they share weight parameters with each other. (e) We curated a series of independent test sets to test the performance of UniMutStab on different tasks. All of them possess a sequence identity of <25% with our training set.

Similarly, the input of UniMutStab consists of nodes, edges, and weights. Specifically, we extract the node features from the general protein pretrained model: ProtT5-XL-Uniref50 [22] is one of the state-of-the-art protein sequence analysis models that leverage the power of the T5 transformer architecture and extensive pretraining on the Uniref50 dataset to provide more accuracy and generalization capabilities than others, such as UniRep [9], EMS-1b^10^, and TAPE [11], for a wide range of bioinformatics tasks. Considering that the trained model has a good learning capability for protein function and structure information, we use it as zero-shot learning and directly extract protein features without further fine-tuning. The output features will be used as the input of the following UniMutStab prediction network. The node *V* is a matrix of N^*^1024, where *N* is the length of the protein and *F*^0^ = 1024 is the size of the ProtT5-XL-Uniref50 last hidden layer. The above approach gets node information for both wild and mutant sequences in Fig. 1b.

As for the edge and weight, we do not obtain them from the experimental or the predicted structure due to our training set is too huge to predict them. We select the protein contact map and its probability predicted by SPOT-Contact-LM [31] as edges (*E*) and weight (*W*).

As shown in Fig. 1c, the corresponding graphs were generated for the wild-type and mutant sequences using the methods mentioned above. A common way to serve as a node through a pretrained model is to take the amino acid node [33], which can only handle a uniform number of mutations (features with fixed dimensions) and will not work for variable numbers of mutations or even insertions and deletions. To be able to handle different mutation types, we put the two graph modules (wild and mutant) into the network separately and share weight to learn from each other during the training process (Fig. 1d). This method will not be limited by the number of mutations and the length of the sequence and can simultaneously learn relevant information about wild and mutant. The output from the previous neural network layer sequentially passes through a linear transformation layer, a normalization layer, a Rectified Linear Unit (ReLU) activation function, and a dropout layer. This process is repeated several times. Finally, we employ a simple linear layer to transform the last layer output into a predicted ΔΔG for the regression task.

We trained UniMutStab on the balanced “Mega-scale” dataset with ~650 000 mutations to ensure our models’ robustness and generalization, where the dataset was split into five groups and each was used once as a test set while the rest served for training. After optimizing the hyperparameters through a 5-fold cross-validation process, we trained the model on the entire data and tested it on several independent datasets. Figure S1 shows that the avg. Pearson correlation coefficient (PCC) and avg. Root-Mean-Square Deviation (RMSE) between the predicted and experimental values are 0.84 and 1.09 kcal/mol, respectively. Another important characteristic of predicting ΔΔG is its antisymmetry, meaning that the Gibbs free energy change for the reverse process (i.e. the free energy change from the wild-type to the mutant form) is equal in magnitude but opposite in sign to that of the direct process: ΔΔG = −ΔΔG. The difference between the direct and the inverse data is only 0.003, which proves that our model essentially helps to learn the ability of antisymmetry property.

### UniMutStab outperforms other state-of-the-art methods on different tasks

UniMutStab is a new tool for predicting the change of protein stability free energy induced by diverse variations. Independent testing is an essential step for comparing the developed model with other methods, including the pretrained model (ESM2), machine learning model, deep learning model, and empirical model. Next, we prepared an extensive series of comparative experiments with existing methods to show the superior performance of our method on single-point, multipoint, indel, and protein design tasks (Fig. 1e). It is worth noting that these datasets have removed the sequences with more than identity 25% with our training set. Some of the latest methods do not provide weights (ProMEP [34]) or feature generation methods (Stability Oracle [35]) and cannot be compared.

#### Evaluating the performances on multiple point mutations

**Figure 2:**
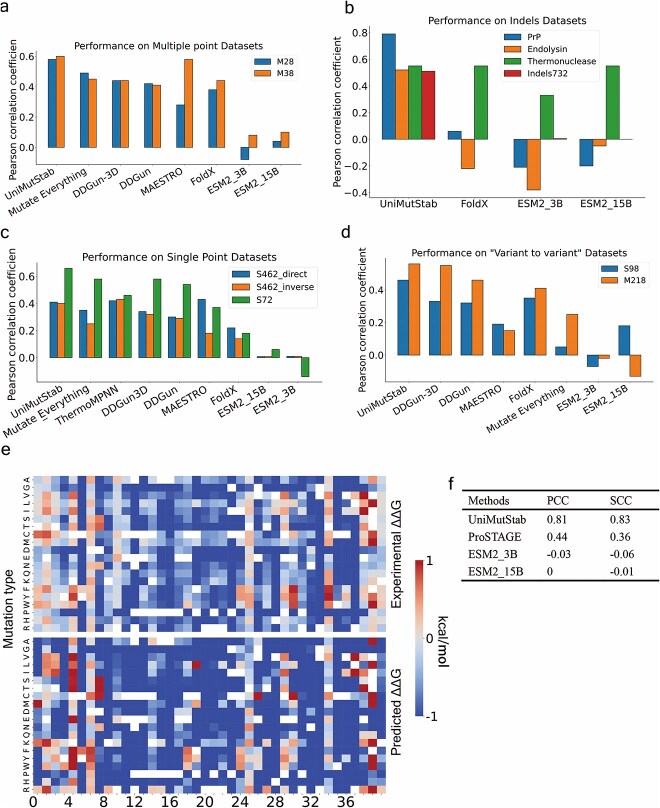
Comparison of UniMutStab with existing SOTA predictors on different tasks. (a) Pearson correlation coefficient on multi-point datasets M28 and M38. The results for the Mutate Everything were obtained on the M38 and M28 test sets, which are missing 12 samples due to the provided code only being able to test two point mutations. (b) Pearson correlation coefficient on indel datasets Prp, Endolysin, Thermonuclease, and Indels732. c. Pearson correlation coefficient on S462 datasets. S462 is obtained by removing the data with a sequence identity of >25% with the training set from S669. It is a dataset widely used to test single-point performance. The “direct” is the original S462, while the “inverse” is the data obtained after reversing the mutation and ΔΔG, which can be used to test the balance of the model. (d) The potential of different predictors for protein engineering was tested on single-point “variant-to-variant” data and multi-point “variant-to-variant” datasets on the S98 and M218 datasets. All original sequences have been mutated. The results for the Mutate Everything were obtained on the M218 test set, which is missing 16 samples. (e) Heat maps of experimental ΔΔG and UniMutStab predicted ΔΔG on a *de novo* designed protein (EHEE_rd2_0487). (f) Pearson and Spearman correlation coefficients between the predicted and measured ΔΔG ​​of UniMutStab, ProSTAGE (the best-performing method on S462), and ESM2 (pretrained model) on the *de novo* designed protein (EHEE_rd2_0487).

Currently, most of the work is developed to predict the thermal stability changes of single-point mutations. Although the predicting effects for single-point mutations are important, in fact, the stability caused by a single mutation may not be sufficient to meet applications in various fields. Only a few methods have been involved in multiple-mutation-induced thermostability changes. One reason for this phenomenon is that the dataset is scarce and difficult to support the development of usable methods; another is the lack of a feasible method. To face the challenge, we used a sequence embedding layer that fuses wild and mutant sequences, which can handle two sequences of any length, and developed it based on a dataset containing tens of thousands of multipoint mutations. We employed two nonredundant blind test sets of M28 and M38 to benchmark it with other available SOTA methods in Fig. 2a.

M28 is a commonly used benchmark set for predicting proteins’ thermal stability with multiple-point mutations. The results indicate that UniMutStab attains impressive values (PCC = 0.58, RMSE = 2.38 compared to all other predictors (with PCC ranging from −0.08 to 0.44, RMSE from 2.49 to 3.01, Fig. 2a). We also tested UniMutStab on the M38 dataset, this blind set was collected from a newly published database. Our result achieves the highest PCC of 0.60. It demonstrates that the performance of UniMutStab is notable in the task of estimating multiple mutations effects for blind datasets and better than other methods.

#### Evaluating the performances on indel mutations

Despite the progress achieved by point substitution prediction, not all innovative aspects of protein evolutionary function have been intensively studied or applied for engineering. Most analyses and methods revolve around amino acid substitutions (single and multiple points), while amino acid insertions and deletions remain overlooked. However, indels are an important source of genetic diversity and have a significant impact on the properties of a protein [36]. We lack a method that is used for assessing the ∆∆G in a fast and accurate way. Here, we use four indel experimental datasets to evaluate the performance of UniMutStab on this task. FoldX [37] is an empirical force field–based method that can predict protein stability, but it requires structures as input, and structures with indel mutations are not easy to get. This is the only method potentially capable of predicting indels, and we try to use it as a benchmark. Compared with FoldX, Fig. 2b indicates the excellent performance of UniMutStab in predicting indel mutations. It achieves PCCs of 0.79, 0.52, 0.55, and 0.51 for PrP, Endolysin, Thermonuclease, and Indels732, respectively. FoldX performs much worse; even endolysin protein predictions show opposite results to the experiment, while our method always maintains acceptable results. In addition, RMSE remains low level compared with FoldX. The ESM2 [23] model is also used to predict the impact of indels but performs poorly too. Our method demonstrates the predictive ability in the field of indels, which will greatly expand the application scope of Artificial intelligence (AI) methods and provides potential methods for more challenging protein engineering and disease prediction in the future.

#### Evaluating the performances on single-point mutations

Prediction of single-point mutations is a classic task, and dozens of methods have been developed, including those based on empirical force fields, machine learning, and deep learning. We additionally validate the ability of UniMutStab to predict changes in stability under single-point mutations. The S669 dataset is a commonly used blind set for fair comparison between existing methods. Similarly, we removed data with a sequence identity >25% with S669 that appeared in any predictor training set and finally retained 46 (69% out of S669). Additionally, we evaluated the inverse dataset of S669, which involves using the mutant sequence as input to predict the ∆∆G when it is reverted to the wild-type sequence. We only show comparable SOTA methods on the S462 direct data, and complete results can be found in Table S5. The results are shown in Fig. 2c, UniMutStab achieves PCC = 0.41 and PCC = 0.40 on S462-direct and S462-inverse, respectively, which is better than all other predictors except ThermoMPNN [38] and is comparable to ThermoMPNN. Furthermore, we conducted verification on the antisymmetry and calculated an *r*_d − i_ value between the predicted direct and inverse mutation results. Our antisymmetry results in a value of *r*_d − i_ = −0.97 and ⟨δ⟩ = 0.07 (Table S5). This means that the model learns information from both direct mutations and inverse mutations. We further evaluated the performance of our model on the newly created dataset, S72, UniMutStab achieves a PCC of 0.66, outperforms all other approaches, and is significantly better than the second-highest method in S462 (ThermoMPNN). Overall, this highlights that the performance of UniMutStab in single-point mutations is more balanced and less biased than all others.

#### Evaluating the performances in the protein engineering field

As described above, all theory-based methods are designed to reduce experimental costs and speed up Research and Development (R&D) progress. Sufficient accuracy is one of the prerequisites for application. Our method has demonstrated excellent results in different tasks. Many high-performance variants in protein stability design are obtained after multiple rounds of mutagenesis [39,40], which means that each round of variants is designed based on the previous round of variants, rather than always based on the wild type. Current evaluations often ignore this important application and only evaluate the impact based on the wild type. Here, we tested the performance of different predictors on single-point “variant to variant” (S98) and multipoint “variant to variant” (M218) datasets, respectively. Both of them are the data of selecting all sequences that already contain mutations in the M38 dataset and mutating them again. In other words, some papers continue to induce mutations after initial sequence variations to further enhance the thermal stability of the protein, All “wild-type” (starting sequence) data contained in these data carry mutations and are divided into single-point and multipoint datasets based on the number of subsequent mutations. It is important to note that in both datasets, the “wild-type” or starting sequences are not natural sequences but rather sequences that have already undergone one or more mutations. Therefore, all the starting sequences in these datasets carry mutations and are further mutated to create the datasets used for testing the predictors. The testing procedure is no different from other datasets. As shown in Fig. 2d, our method achieves a PCC of 0.46 and an RMSE of 1.25 kcal/mol on S98; all other methods perform much worse. On the M218, our method still achieves the best results, in which the PCC is 0.56 and RMSE is 2.01 kcal/mol. Another challenging task is the engineering of *de novo* proteins. The latest AI methods can write protein sequences from scratch without starting from natural sequences [41], which brings new challenges to stability prediction tasks. It is difficult to obtain sufficient evolutionary information through multiple sequence alignment methods for *de novo* designed sequences, leading to the failure/performance degradation of Multiple Sequence Alignment based (MSA-based) methods. To further elucidate the implications of UniMutStab predictions, we applied our model to predict protein thermodynamic stability changes for missense variants on *de novo* designed proteins. EHEE_rd2 is a protein sequence designed by Tsuboyama *et al*. [24], which is 40aa in length. We visualized the predicted ∆∆G of the EHEE_rd2_0487 (<25% sequence identity with training set) saturation mutation and compared it with the resulting score of the ESM2 model (Fig. 2e and f). It is worth noting that the degree of agreement between the predictions of UniMutStab and the measured ΔΔG values is significantly higher than that of ESM2 and even better than ProSTAGE [11] (the best-performing method in S462, Table S5). This phenomenon is also confirmed by the PCC in Fig. 2f (UniMutStab 0.80 versus ProSTAGE 0.44 versus ESM2_3B -0.03), implying a closer relation between UniMutStab predicted values and protein stability. Therefore, UniMutStab demonstrates the ability to deeply optimize protein sequences and provides an economically feasible solution for developing high-performance enzymes.

### Zero-shot learning for protein stability analysis

The ability to predict stability changes resulting from insertion and deletion mutations opens up doors for assessing a wider range of applications. The application of zero-shot learning in our method is pivotal for assessing its ability to generalize across unseen tasks and datasets. UniMutStab was deployed in zero-shot tasks, leveraging a comprehensive bacterial OGT dataset, along with mutation-related disease datasets sourced from ClinVar. By rigorously evaluating protein stability across a diverse array of bacterial species and elucidating the stability–pathogenicity relationship in genetic mutations, our approach has demonstrated significant generalization capabilities.

#### Evaluation of the generalization performance of UniMutStab with bacterial optimal growth temperature dataset

**Figure 3:**
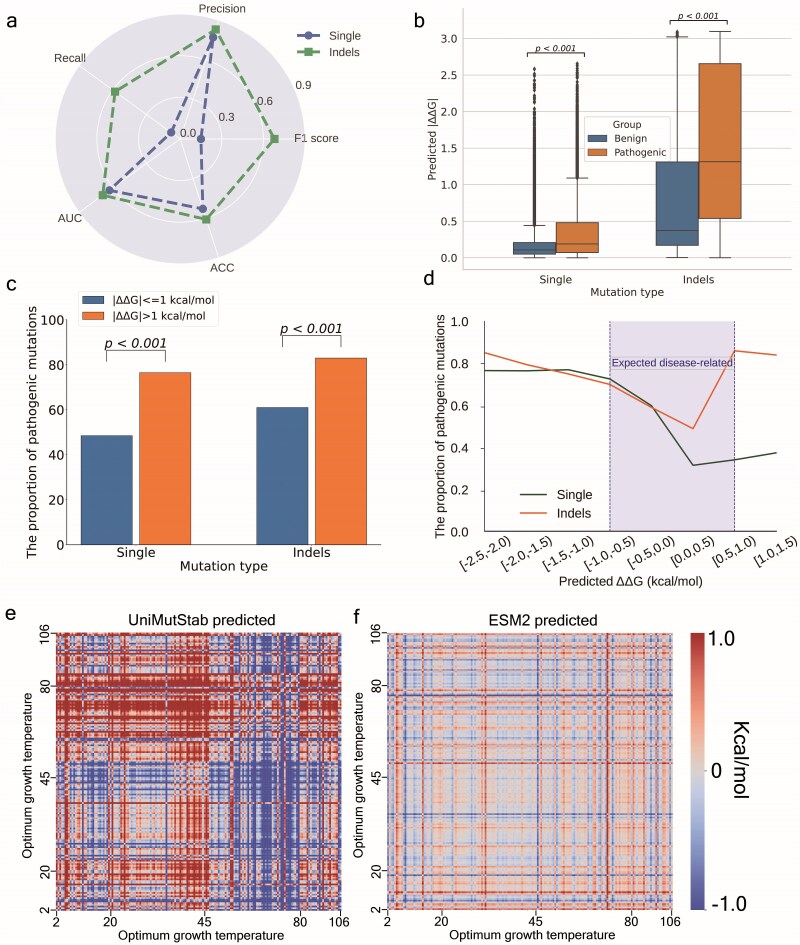
Explore the performance of UniMutStab on zero-shot prediction tasks. (a) Predicted ΔΔG values ​​are used to distinguish pathogenic from benign variants. The radar plot shows the AUC, recall, precision, accuracy, and F1 metrics for single and indel prediction. (b) Average values ​​of predicted |∆∆G| for pathogenic and benign mutations in single and indel mutations. (c) The proportion of pathogenicity among single point and indel mutations when a variant with |∆∆G| ≤1 kcal/mol is benign and |∆∆G| >1 kcal/mol is pathogenic. (d) The distribution of the proportion of pathogenic mutations on the predicted |∆∆G|. Heatmap of ∆∆G predicted by UniMutStab (e) and ESM2 (f) on the optimal growth temperature dataset of bacteria.

Organisms exhibit varying growth rates at different temperatures. At their OGT, organisms maintain the best rates of various reactions within their bodies, and this maintenance is heavily reliant on the support of proteins. Thermophiles express proteins that are highly stable and rigid to withstand high temperatures, while psychrophiles express proteins that are more flexible to function efficiently at low temperatures. Mesophiles express proteins with stability characteristics that are suitable for moderate temperatures [42]. Proteins in species with different optimal growth temperatures have evolved distinct stabilities to function effectively across various environments. One of the key advantages of our method is its ability to evaluate the stability differences between various sequences, thereby offering a robust framework to assess the generalization performance of our approach. In this context, we compiled a comprehensive bacterial optimal growth temperature dataset that includes a wide range of bacterial species, covering the optimal growth temperatures of psychrophilic (cold-loving) to thermophilic (heat-loving) organisms, as well as the proteins they express. In the dataset, there are 14 987 proteins with 340 psychrophiles (*T*_opt_ < 20°C), 12 853 mesophiles (20 ≤ *T*_opt_ < 45°C), 1511 thermophiles (45 ≤ *T*_opt_ < 80°C), and 283 hyperthermophiles (80°C ≤ *T*_opt_). We use a heat map in Fig. 3e and f to show the predicted ∆∆Gs between all proteins in the OGT dataset. Thermophilic proteins are expected to exhibit decreased stability (indicated in blue) upon evolving into other proteins, whereas psychrophilic proteins are anticipated to show increased stability (indicated in red) following similar evolutionary changes. When the diagonal line is used as a dividing axis, the left side of the graph is supposed to display red, while the right side is supposed to display blue. Our results indicate that a few regions deviate from our expectations; for example, the anomalous region is predicted to be green, whereas it should be red. One is the region where thermophilic bacterial proteins are mutated to hyperthermophilic bacterial proteins. A possible explanation for this discrepancy is that while most thermophilic proteins are thermostable, this is not universally the case [43]. Adaptation to high temperatures may also be facilitated by other mechanisms, such as rapid protein synthesis and degradation, as well as the assistance of molecular chaperones [44]. Another anomalous region is where psychrophilic bacterial proteins are mutated to mesophilic proteins, which could be due to test data noise or errors. In summary, these incorrect predictions may be related to evolutionary, adaptive mechanisms, and data errors. Nevertheless, overall, our results demonstrate a marked improvement over those predicted by ESM2_3B. The OGT result shows that UniMutStab has good generalization across species and is helpful for understanding the evolution of species [45] and discovering potential industrial enzyme candidates. It also provides a robust benchmark for evaluating the accuracy and reliability of computational methods used to predict protein stability.

#### Explore the application of UniMutStab in mutation-related diseases

Changes in protein stability are reflected in structural folding, and it has been reported in the literature that protein misfolding will lead to the formation of insoluble fibrillar aggregates that hinder/loss of normal function, which is generally the main consequence of pathogenic mutations [46,47]. However, the amount of validating experiments is still negligible as compared to human diseases. In this work, we try to understand and analyze pathogenic variants from the perspective of protein stability by using UniMutStab. The bigger the change in stability value, the more likely it is to cause disruptive effects on the structure. According to the previous works [46,48,49], we set the |∆∆G| threshold as 1 kcal/mol to classify the pathogenic and benign. In this definition, a variant with |∆∆G| ≤1 kcal/mol is benign, while ∆∆G > 1 kcal/mol with increasing protein stability and ∆∆G < −1 kcal/mol with decreasing protein stability are both supposed to be pathogenic. The disease-related datasets were downloaded from the ClinVar database, which contains 21 192 single-point mutation data and 1508 insertion/deletion/duplication data. Our method performs better in distinguishing indels from pathogenic mutations than single-point mutations, indicating that the association between stability and pathogenicity of the two types of mutations is mechanistically different (Fig. 3a). Nevertheless, the precision of single points and indels still reaches 0.77 and 0.83, respectively, especially the F1 reaches 0.68 on the indels dataset. As shown in Fig. 3b, compared with pathogenic mutations, benign mutations have smaller |∆∆G|, which means that ClinVar disease-related variations in proteins generally promote a much larger effect on protein stability than polymorphisms on the same proteins, also confirming that stability perturbation plays a crucial role in impairing protein function [48]. In both the single and indels datasets, large |∆∆G| values are associated with a higher likelihood of causing disease, whereas small |∆∆G| values are associated with a lower likelihood (Fig. 3c). Figure 3d shows the pathogenicity of different |∆∆G| intervals. Surprisingly, two types of mutations show the same trend when |∆∆G| ≤0, i.e. the larger the |∆∆G|, the higher the pathogenicity. However, when |∆∆G| >0, single-point mutations still keep low pathogenicity, while the pathogenicity of indels increases rapidly. This phenomenon is also consistent with the different average mutation rates of single points and indels in Fig. 3c. One possible reason is that indels are destructive to protein function, while single point mutations tend to only change the thermal stability of the protein. The larger |∆∆G| exhibited by indels may provide an attractive way to engineer industrial enzymes with surprising performance.

### Ablation experiment of UniMutStab

**Figure 4:**
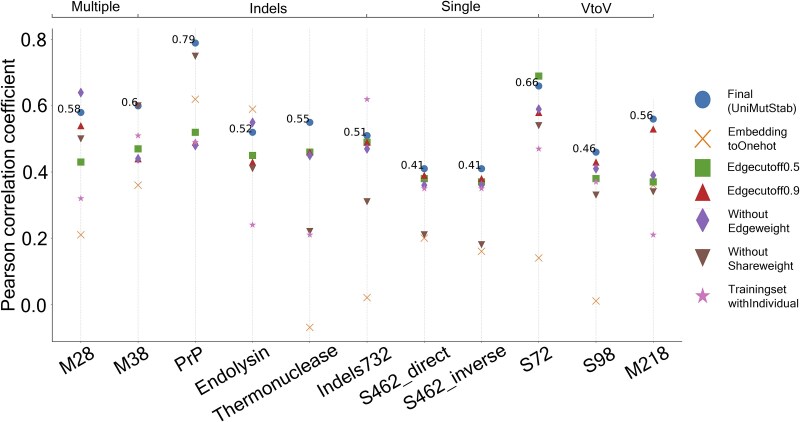
Performance comparison of UniMutStab trained on different modules. Results on various tasks show that the final model has significantly better generalization capabilities than the other model that excludes a certain module. Trainingset ablation experiments mean that we first train the models on datasets containing only one type of mutation. Then, we use these models to test the corresponding datasets.

Features from multiple sources constitute UniMutStab. To assess the necessity of each component, we performed ablation experiments by excluding sequence embedding or other key components from the well-established UniMutStab. Tables S9–S11 show the validation performance of the defective variants of UniMutStab in predicting protein stability and demonstrate that all components are essential for the final model. Specifically, node features are encoded using one-hot encoding instead of protein embeddings, contact graphs are processed using different thresholds, or edge features/shared weight are excluded. In addition, we tested the performance of UniMutStab on all of the datasets with GraphConv, SAGEConv, GINConv, GATConv, and ChebConv, resulting in SAGEConv achieving the best performance. Initially, we observed suboptimal outcomes when training on each dataset individually. In Fig. 4, we discovered that training the model on a combined training dataset of single-point, multipoint, and indel mutations yielded significantly better performance compared to training on these datasets separately. The combined dataset provided a more diverse and comprehensive set of mutation scenarios, allowing the model to capture a broader range of patterns and interactions. This approach led to marked improvements in prediction accuracy and model stability. Key reasons may include increased data diversity, shared underlying principles among different mutation types, and reduced overfitting. Additionally, UniMutStab significantly outperforms models trained on unbalanced datasets, indicating that balancing the dataset helps improve the model’s generalization ability (Tables S2–S8). The complete dataset also helps improve the performance of the model (Fig. S2). These factors collectively contributed to the enhanced performance observed in our experiments.

## Discussion

Protein thermodynamic prediction is a critical aspect of understanding protein stability, folding, and function [1, 2]. The ability to accurately predict thermodynamic properties such as folding free energy, stability under various conditions, and the impact of mutations is essential for a wide range of applications in biochemistry, molecular biology, and biotechnology. Traditional models primarily focus on single-point mutations and typically employ machine learning or deep learning approaches to predict the impact of these single-point mutations on protein stability. The core principle involves extracting features related to single-point mutations and using these features in machine learning or deep learning models to predict the effect of protein mutations on stability. However, multipoint mutations and indel mutations present a significant challenge due to the variable number of amino acid changes, which makes it difficult to use conventional machine learning or deep learning methods that require fixed-length features. Although techniques like averaging or padding can be used to achieve consistent feature lengths, these methods often obscure important details, especially when the number of mutations is relatively small. Nevertheless, existing methods are still in their infancy of multiple and indels evaluation and new methods need to be developed to address more challenges. Here, we describe a new deep learning approach for predicting protein thermostability changes based on sequence and residue contact map representations. Our method, UniMutStab, is trained on massive sequences from the new “Mega-scale” dataset and rapidly predicts all types of mutations ∆∆G of proteins and improves over existing sequence/structure-based methods on the majority of application scenarios. Features learned from sequence embeddings by the ProtT5-XL-Uniref50 model and amino acid contact maps by the GCN result in substantial improvements in protein stability prediction, thereby leading to novel applications. Although pretrained models claim to have achieved success in various downstream tasks, they cannot actually be applied to protein stability prediction (as evidenced by their poor performance on the zero-shot benchmark) because they have difficulty capturing subtle differences. Therefore, one important advantage of UniMutStab is its ability to predict the stability differences of unknown proteins by extracting wild-type and mutant sequence features. In addition, the cost time in Table S12 shows that our method can complete a prediction task (500aa) in just 4 s, and it is expected to achieve genome-level prediction tasks through parallel technology. By comparing the prediction performance of different types of mutations, we demonstrate that UniMutStab has a wide range of applications. The robustness of sequence-based methods suggests that their ability to use sequence embeddings opens the door to characterizing many proteins that lack experimentally determined structures. Although this paper focuses on models for predicting protein stability, it also provides cases of its application. We analyze the role of stability in the diseases caused by mutations and the relationship between stability and species adaptability. In conclusion, these predictions can provide insights into the fundamental principles of protein folding, guide the design of stable proteins for industrial and therapeutic use, and assist in the interpretation of experimental data. Furthermore, understanding the thermodynamics of protein is crucial for drug design and the development of therapeutic proteins. Despite the complexity and challenges involved, advancements in computational methods, along with a growing body of experimental data, are enhancing our ability to make reliable thermodynamic predictions. This progress holds promise for significant breakthroughs in protein engineering, disease treatment, and the development of novel biomaterials.

**Key Points**

- Fully sequence-based approaches extend the application to the whole genome, avoiding limitations due to missing or low-quality predicted structures.
- Building a general stability prediction framework that can deal with any mutation types.
- Designing an innovative shared-graph convolutional network to exploit the synergistic effect of protein language models and residue interaction networks.
- Evaluating the predictive performance of the proposed method comprehensively and comparing it with existing state-of-the-art methods fairly by using a series of benchmark datasets.
- Discussing the potential implications of the findings on protein engineering strategies and the understanding of pathogenic mutation mechanisms by deeply analyzing the result of zero-shot tasks.

## Supplementary Material

SI_R2_bbaf190

## Data Availability

All the datasets and analysis code can be found in https://github.com/GenScript-IBDPE/UniMutStab.
