# Supplementary material for: Shared-weight graph framework for comprehensive protein stability prediction across diverse mutation types
Source: Brief Bioinform. 2025 Apr 24;26(2):bbaf190. doi: 10.1093/bib/bbaf190 (PMC12021015; doi:10.1093/bib/bbaf190)
Supplement: SI_R2_bbaf190 [file si_r2_bbaf190.docx]

Shared-weight Graph Framework for Comprehensive Protein Stability Prediction across Diverse Mutation Types

*Gen Li^a^*^†^*, Sijie Yao^a^*^†^ *and Long Fan^a*^*

^a^Production and R&D Center I of LSS, GenScript (Shanghai) Biotech Co.,Ltd., Shanghai, 200131, China

^†^These authors contributed equally to this work.

*To whom correspondence should be addressed. Email: leo.fan@genscript.com

Table S1. Datasets used in this work.

| Source  name | Name for this work | Used for | Sample number | Protein number | Source  Doi Link |
| --- | --- | --- | --- | --- | --- |
| Mega-scale | Traing set | Single | 163998 | 158 | 10.1038/s41586-023-06328-6 |
|  |  | Multiple | 127542 | 125 |  |
|  |  | Indels | 34368 | 193 |  |
| S669 | S462 | Single point mutation blind test set | 462 | 73 | 10.1093/bib/bbab555 |
| S96 | S72 | Single point mutation blind test | 72 | 11 | 10.1093/nar/gkac325 |
| M28 | M28 | Multiple point mutation blind test | 28 | 12 | 10.1093/nar/gkac325 |
| M38 | M38 | Multiple point mutation blind test | 38 | 13 | MPTherm, ProthermDB, ThermoMutDB and FireProtDB |
| S98 | S98 | Single point mutation blind test | 98 | 3 | is the data of selecting sequences that already contain mutations in the M38 dataset and mutating them again |
| M218 | M218 | Multiple point mutation blind test | 218 | 6 | is the data of selecting sequences that already contain mutations in the M38 dataset and mutating them again |
| P00720 | Endolysin | Insertion and deletion mutation blind test | 22 | 1 | 10.1006/jmbi.1994.1195 |
| P04925 | PrP | Insertion and deletion mutation blind test | 12 | 1 | 10.1021/bi982714g |
| P00644 | Thermonuclease | Insertion and deletion mutation blind test | 4 | 1 | 10.1002/prot.10216 |
| Indels732 | Indels732 | Insertion and deletion mutation blind test | 732 | 379 | Indels732 is a test set of 732 insertion/deletion samples selected from cDNA data that comply with the rule of less than 25% similarity with the training set. |


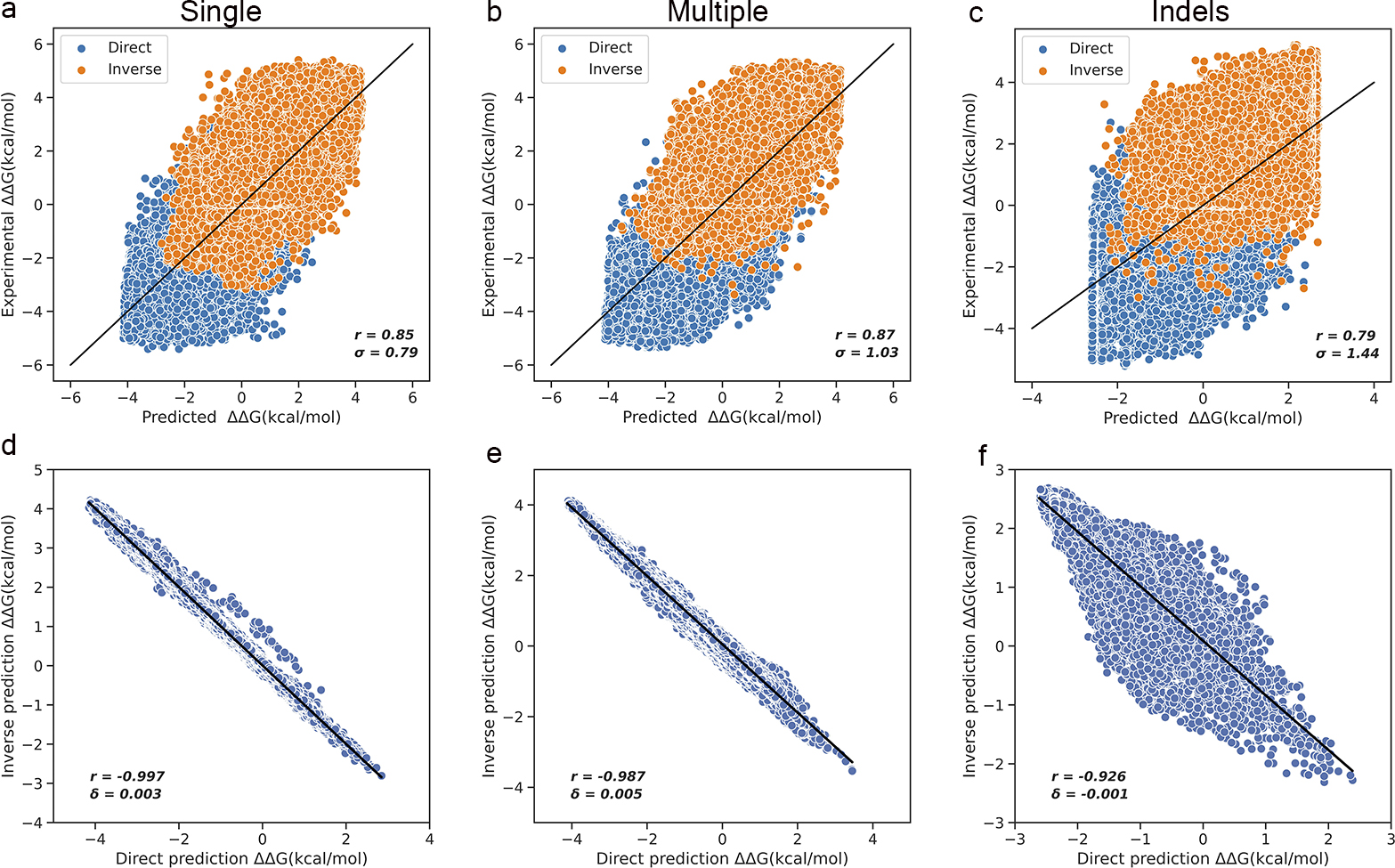


Figure S1. Five-fold cross validation result of the training dataset. (a, b, c) The overall Pearson correlation coefficient (PCC, r) and RMSE (σ) of direct (orange) and inverse (blue) predictions on different mutation types. (d, e, f) The r and bias (δ) between direct and inverse predictions.

Table S2. Performance comparison of UniMutStab with other predictors on M28 set

| Methods | PCC | RMSE | MAE | SCC |
| --- | --- | --- | --- | --- |
| DDGun | 0.42 | 2.49 | 1.92 | 0.43 |
| DDGun3D | 0.44 | 2.54 | 1.96 | 0.46 |
| FoldX | 0.38 | 2.64 | 2.01 | 0.41 |
| MAESTRO | 0.28 | 2.90 | 2.33 | 0.12 |
| ESM2_3B | -0.08 | 2.98 | 2.45 | -0.05 |
| ESM2_15B | 0.04 | 3.01 | 2.44 | 0.09 |
| UniMutStab_unbalance | 0.42 | 2.36 | 1.91 | 0.47 |
| UniMutStab** | 0.58 | 2.38 | 1.82 | 0.53 |

“**” indicates a highly statistically significant (p < 0.01), and “*” indicates a statistically significant (p < 0.05).

Table S3. Performance comparison of UniMutStab with other predictors on M38 set

| Methods | PCC | RMSE | MAE | SCC |
| --- | --- | --- | --- | --- |
| DDGun | 0.41 | 2.51 | 1.12 | 0.42 |
| DDGun-3D | 0.44 | 2.60 | 2.21 | 0.45 |
| FoldX | 0.44 | 2.37 | 1.88 | 0.44 |
| MAESTRO | 0.58 | 1.92 | 1.48 | 0.61 |
| ESM2_3B | 0.08 | 3.03 | 2.53 | 0.16 |
| ESM2_15B | 0.10 | 3.04 | 2.55 | 0.02 |
| UniMutStab_unbalance | 0.68 | 2.03 | 1.55 | 0.71 |
| UniMutStab** | 0.60 | 2.46 | 2.04 | 0.66 |

“**” indicates a highly statistically significant (p < 0.01), and “*” indicates a statistically significant (p < 0.05).

Table S4. Comparison of the results on the indels datasets.

| Protein | Methods | PCC | RMSE | MAE | SCC |
| --- | --- | --- | --- | --- | --- |
| PrP | FoldX | 0.06 | 210 | 210 | 0.08 |
|  | UniMutStab_unbalance | 0.54 | 2.11 | 1.92 | 0.06 |
|  | UniMutStab** | 0.79 | 1.17 | 0.97 | 0.72 |
|  | ESM2_3B | -0.21 | 1.37 | 1.11 | -0.28 |
|  | ESM2_15B | -0.20 | 1.69 | 1.03 | 0.12 |
| Endolysin | FoldX | -0.22 | 7.9 | 6.02 | -0.20 |
|  | UniMutStab_unbalance | 0.47 | 1.22 | 1.03 | 0.42 |
|  | UniMutStab* | 0.52 | 2.29 | 2.09 | 0.52 |
|  | ESM2_3B | -0.38 | 2.76 | 2.55 | -0.30 |
|  | ESM2_15B | -0.05 | 2.79 | 2.58 | -0.06 |
| Thermonuclease | FoldX | 0.55 | 4.38 | 3.54 | 0.4 |
|  | UniMutStab_unbalance | 0.29 | 1.35 | 1.28 | 0 |
|  | UniMutStab | 0.55 | 1.29 | 1.24 | 0.80 |
|  | ESM2_3B | 0.33 | 0.76 | 0.63 | 0.40 |
|  | ESM2_15B | 0.55 | 0.70 | 0.61 | 0.40 |
|  | UniMutStab_unbalance | 0.08 | 0.94 | 0.72 | 0.53 |
| Indels732 | UniMutStab** | 0.51 | 0.94 | 0.65 | 0.70 |
|  | ESM2_3B | 0 | 2.04 | 1.76 | 0 |
|  | ESM2_15B | 0 | 2.08 | 1.78 | 0.01 |

“**” indicates a highly statistically significant (p < 0.01), and “*” indicates a statistically significant (p < 0.05).

Table S5. Comparison of UniMutStab with existing predictiors on S462 dataset.

| Methods | S462_direct | | | | S462_inverse | | | | | Antisimmetry/Bias | | |  |
| --- | --- | --- | --- | --- | --- | --- | --- | --- | --- | --- | --- | --- | --- |
|  | PCC | RMSE | MAE | SCC | | PCC | RMSE | MAE | SCC | | *r*_d-i_ | bias | |
| INPS-Seq | 0.37 | 1.66 | 1.21 | 0.36 | | 0.36 | 1.67 | 1.21 | 0.35 | | -1 | 0 | |
| ACDC-NN | 0.38 | 1.65 | 1.18 | 0.36 | | 0.37 | 1.67 | 1.19 | 0.34 | | 0.98 | 0.02 | |
| PremPS | 0.32 | 1.68 | 1.23 | 0.31 | | 0.33 | 1.67 | 1.21 | 0.32 | | -0.8 | 0.08 | |
| ACDC-NN-Seq | 0.34 | 1.68 | 1.21 | 0.33 | | 0.34 | 1.68 | 1.21 | 0.33 | | -1 | 0 | |
| INPS3D | 0.35 | 1.68 | 1.21 | 0.35 | | 0.31 | 1.86 | 1.38 | 0.33 | | -0.48 | -0.40 | |
| DDGun3D | 0.34 | 1.78 | 1.26 | 0.31 | | 0.32 | 1.80 | 1.29 | 0.31 | | -0.96 | -0.04 | |
| DDGun | 0.30 | 1.92 | 1.38 | 0.30 | | 0.29 | 1.95 | 1.40 | 0.3 | | -0.95 | -0.03 | |
| ThermoNet | 0.30 | 1.77 | 1.30 | 0.28 | | 0.30 | 1.79 | 1.33 | 0.26 | | -0.84 | -0.04 | |
| Dynamut | 0.34 | 1.74 | 1.31 | 0.29 | | 0.28 | 1.83 | 1.35 | 0.3 | | -0.56 | -0.06 | |
| MAESTRO | 0.43 | 1.59 | 1.19 | 0.39 | | 0.18 | 2.16 | 1.7 | 0.18 | | -0.17 | -0.57 | |
| PopMusic | 0.34 | 1.68 | 1.22 | 0.33 | | 0.21 | 2.16 | 1.71 | 0.19 | | -0.3 | -0.72 | |
| DUET | 0.33 | 1.69 | 1.25 | 0.32 | | 0.23 | 2.21 | 1.73 | 0.26 | | -0.08 | -0.69 | |
| mCSM | 0.28 | 1.71 | 1.27 | 0.28 | | 0.22 | 2.33 | 1.88 | 0.23 | | 0 | -0.86 | |
| I-Mutant3.0-Seq | 0.26 | 1.72 | 1.29 | 0.26 | | 0.19 | 2.24 | 1.79 | 0.23 | | -0.46 | -0.72 | |
| I-Mutant3.0 | 0.30 | 1.67 | 1.24 | 0.28 | | 0.09 | 2.38 | 1.90 | 0.11 | | 0.03 | -0.84 | |
| FoldX | 0.22 | 2.35 | 1.63 | 0.28 | | 0.14 | 2.81 | 1.73 | 0.25 | | -0.20 | -0.47 | |
| SDM | 0.33 | 1.81 | 1.39 | 0.33 | | 0.11 | 2.26 | 1.73 | 0.11 | | -0.44 | -0.41 | |
| SAAFEC-SEQ | 0.30 | 1.67 | 1.24 | 0.28 | | 0.01 | 2.38 | 1.91 | 0.02 | | -0.07 | -0.83 | |
| Dynamut2 | 0.23 | 1.75 | 1.31 | 0.25 | | 0.14 | 2.24 | 1.77 | 0.08 | | 0.16 | -0.62 | |
| MuPro | 0.15 | 1.78 | 1.34 | 0.15 | | 0.16 | 2.39 | 1.94 | 0.17 | | -0.27 | -0.93 | |
| PROST | 0.37 | 1.64 | 1.16 | 0.35 | | 0.38 | 1.64 | 1.18 | 0.38 | | 0.88 | 0.04 | |
| PROSTATA | 0.42 | 1.59 | 1.12 | 0.42 | | 0.42 | 1.59 | 1.13 | 0.42 | | -0.99 | -0.01 | |
| ThermoMPNN | 0.42 | 1.59 | 1.11 | 0.47 | | 0.43 | 1.65 | 1.19 | 0.48 | | -0.88 | -0.21 | |
| ProSTAGE | 0.52 | 1.49 | 1.08 | 0.51 | | 0.50 | 1.52 | 1.12 | 0.47 | | -0.89 | 0.01 | |
| MutateEverything | 0.35 | 1.64 | 1.17 | 0.34 | | 0.25 | 2.00 | 1.55 | 0.25 | | -0.16 | 0.46 | |
| Stability-Oracle^*^ | 0.43 | 1.67 | 1.23 | 0.43 | | 0.42 | 1.61 | 1.16 | 0.43 | | -0.99 | -0.13 | |
| ESM2_3B | 0.03 | 1.43 | 0.94 | -0.03 | | 0.03 | 1.43 | 0.94 | -0.03 | | -1 | 0 | |
| ESM2_15B | -0.10 | 1.44 | 0.95 | -0.04 | | 0.03 | 1.43 | 0.94 | -0.03 | | -1 | 0 | |
| UniMutStab_unbalance | 0.20 | 1.71 | 1.29 | 0.10 | | 0.32 | 2.35 | 1.93 | 0.37 | | -0.25 | -0.88 | |
| UniMutStab** | 0.41 | 1.73 | 1.29 | 0.44 | | 0.40 | 1.69 | 1.24 | 0.41 | | -0.97 | 0.07 | |

The RMSE, and MAE values for direct and inverse mutations are expressed in kcal/mol. The Pearson correlation coefficient *r*_d−i_ between the predicted ΔΔG values of direct and inverse mutations, and the bias are listed. Results are taken from Pancotti et al. Stability-Oracle removed 3 samples because the training set overlapped with S462. UniMutStab_unbalance means that our model is trained on the unbalanced original dataset. “**” indicates a highly statistically significant (p < 0.01), and “*” indicates a statistically significant (p < 0.05).

Table S6. Comparison of UniMutStab with existing predictors on S72 dataset

| Methods | PCC | RMSE | MAE | SCC |
| --- | --- | --- | --- | --- |
| DDGun | 0.54 | 2.22 | 1.61 | 0.50 |
| DDGun3D | 0.58 | 2.16 | 1.63 | 0.53 |
| FoldX | 0.18 | 4.48 | 2.58 | 0.35 |
| MAESTRO | 0.37 | 2.43 | 1.72 | 0.34 |
| INPS-MD | 0.48 | 2.34 | 1.75 | 0.43 |
| mCSM | 0.34 | 2.49 | 1.83 | 0.41 |
| INPS-Seq | 0.48 | 2.31 | 1.70 | 0.45 |
| PopMusic | 0.35 | 2.50 | 1.90 | 0.32 |
| SDM | 0.51 | 2.25 | 1.67 | 0.50 |
| PROSTATA | 0.50 | 2.31 | 1.65 | 0.47 |
| ThermoMPNN | 0.46 | 2.35 | 1.75 | 0.45 |
| MutateEverything | 0.58 | 2.25 | 1.56 | 0.55 |
| Stability-Oracle* | -0.07 | 2.03 | 1.71 | 0.10 |
| ESM2_3B | -0.14 | 2.71 | 2.04 | -0.21 |
| ESM2_15B | 0.13 | 2.70 | 2.04 | 0.12 |
| UniMutStab_unbalance | 0.52 | 2.53 | 1.86 | 0.42 |
| UniMutStab** | 0.66 | 2.45 | 1.87 | 0.54 |

Only the methods that perform well on S462 are selected for testing S72 dataset. “*” Some data cannot be obtained due to incomplete open source code. “**” indicates a highly statistically significant (p < 0.01), and “*” indicates a statistically significant (p < 0.05).

Table S7. Comparison of UniMutStab with existing predictors on S98 set

| Methods | PCC | RMSE | MAE | SCC |
| --- | --- | --- | --- | --- |
| MAESTRO | 0.19 | 1.80 | 1.50 | 0.16 |
| FoldX | 0.35 | 1.94 | 1.16 | 0.36 |
| DDGun | 0.32 | 2.03 | 1.61 | 0.33 |
| DDGun3D | 0.33 | 1.41 | 1.13 | 0.35 |
| ThermoMPNN | 0.35 | 1.44 | 1.19 | 0.34 |
| MutateEverything | 0.05 | 1.77 | 1.44 | 0.03 |
| ESM2_3B | -0.07 | 1.39 | 1.19 | -0.04 |
| ESM2_15B | 0.18 | 1.37 | 1.16 | 0.15 |
| UniMutStab_unbalance | 0.36 | 1.85 | 1.50 | 0.34 |
| UniMutStab** | 0.46 | 1.25 | 1.06 | 0.43 |

PROSTATA training set includes the S98 dataset. “**” indicates a highly statistically significant (p < 0.01), and “*” indicates a statistically significant (p < 0.05).

Table S8. Performance comparison of UniMutStab with other predictors on M218 set

| Methods | PCC | RMSE | MAE | SCC |
| --- | --- | --- | --- | --- |
| MAESTRO | 0.15 | 2.90 | 2.34 | 0.14 |
| Foldx | 0.41 | 2.91 | 2.26 | 0.40 |
| DDGun | 0.46 | 2.04 | 1.63 | 0.37 |
| DDGun-3D | 0.55 | 1.96 | 1.57 | 0.46 |
| ESM2_3B | -0.02 | 2.30 | 1.82 | -0.04 |
| ESM2_15B | -0.13 | 2.33 | 1.85 | -0.15 |
| UniMutStab_unbalance | 0.30 | 2.60 | 2.08 | 0.31 |
| UniMutStab** | 0.56 | 2.01 | 1.60 | 0.52 |

“**” indicates a highly statistically significant (p < 0.01), and “*” indicates a statistically significant (p < 0.05).

Table S9. Ablation study of UniMutStab on multiple point mutations.

| ID | Model | Features | | | | M28 | | M38 | | M218 | |
| --- | --- | --- | --- | --- | --- | --- | --- | --- | --- | --- | --- |
|  |  | Node features | Contact Map Cutoff | Edge | Shared  Weight | PCC | RMSE | PCC | RMSE | PCC | RMSE |
| 1 | SageConv （UniMutStab） | T5 embedding | 0.1 | Yes | Yes | 0.58 | 2.38 | 0.60 | 2.46 | 0.56 | 2.01 |
| 2 | SageConv | Onehot | 0.1 | Yes | Yes | 0.21 | 2.42 | 0.36 | 2.21 | 0.36 | 2.27 |
| 3 | SageConv | T5 embedding | 0.1 | No | Yes | 0.64 | 2.11 | 0.44 | 2.31 | 0.39 | 2.15 |
| 4 | SageConv | T5 embedding | 0.9 | Yes | Yes | 0.54 | 2.26 | 0.44 | 2.33 | 0.53 | 1.97 |
| 5 | SageConv | T5 embedding | 0.5 | Yes | Yes | 0.43 | 2.64 | 0.47 | 2.60 | 0.37 | 2.15 |
| 6 | SageConv | T5 embedding | 0.05 | Yes | Yes | 0.56 | 2.39 | 0.45 | 2.60 | 0.30 | 2.19 |
| 7 | GATConv | T5 embedding | 0.1 | Yes | Yes | 0.58 | 2.32 | 0.47 | 2.29 | 0.30 | 2.25 |
| 8 | GraphConv | T5 embedding | 0.1 | Yes | Yes | 0.43 | 2.61 | 0.50 | 2.47 | 0.47 | 2.06 |
| 9 | ChebConv | T5 embedding | 0.1 | Yes | Yes | 0.55 | 2.33 | 0.46 | 2.48 | 0.54 | 1.99 |
| 10 | GINConv | T5 embedding | 0.1 | Yes | Yes | 0.46 | 2.54 | 0.47 | 2.53 | 0.26 | 2.25 |
| 11 | SageConv (Individual) | T5 embedding | 0.1 | Yes | Yes | 0.32 | 2.89 | 0.51 | 2.58 | 0.21 | 2.27 |
| 12 | SageConv | T5 embedding | 0.1 | Yes | No | 0.50 | 2.25 | 0.55 | 2.24 | 0.34 | 2.26 |

Table S10. Ablation study of UniMutStab on indels mutations.

| ID | Model | Features | | | | PrP | | Endolysin | | Thermonuclease | | Indels732 | |
| --- | --- | --- | --- | --- | --- | --- | --- | --- | --- | --- | --- | --- | --- |
|  |  | Node features | Contact Map Cutoff | Edge | Shared  Weight | PCC | RMSE | PCC | RMSE | PCC | RMSE | PCC | RMSE |
| 1 | SageConv （UniMutStab） | T5 embedding | 0.1 | Yes | Yes | 0.79 | 1.17 | 0.52 | 2.29 | 0.55 | 1.29 | 0.51 | 0.94 |
| 2 | SageConv | Onehot | 0.1 | Yes | Yes | 0.62 | 2.01 | 0.59 | 1.71 | -0.07 | 0.55 | 0.02 | 1.22 |
| 3 | SageConv | T5 embedding | 0.1 | No | Yes | 0.48 | 1.90 | 0.55 | 1.91 | 0.45 | 1.78 | 0.47 | 0.91 |
| 4 | SageConv | T5 embedding | 0.9 | Yes | Yes | 0.49 | 1.70 | 0.43 | 2.04 | 0.46 | 1.60 | 0.49 | 1.01 |
| 5 | SageConv | T5 embedding | 0.5 | Yes | Yes | 0.52 | 1.20 | 0.45 | 2.37 | 0.46 | 1.86 | 0.49 | 0.94 |
| 6 | SageConv | T5 embedding | 0.05 | Yes | Yes | 0.76 | 1.51 | 0.61 | 2.32 | 0.47 | 1.63 | 0.49 | 0.96 |
| 7 | GATConv | T5 embedding | 0.1 | Yes | Yes | 0.66 | 1.53 | 0.47 | 2.39 | 0.34 | 1.62 | 0.46 | 1.08 |
| 8 | GraphConv | T5 embedding | 0.1 | Yes | Yes | 0.58 | 1.42 | 0.54 | 2.30 | 0.28 | 1.76 | 0.49 | 0.97 |
| 9 | ChebConv | T5 embedding | 0.1 | Yes | Yes | 0.67 | 1.01 | 0.55 | 2.15 | 0.41 | 1.84 | 0.49 | 0.98 |
| 10 | GINConv | T5 embedding | 0.1 | Yes | Yes | 0.56 | 1.11 | 0.10 | 2.86 | 0.59 | 1.42 | 0.41 | 1.10 |
| 11 | SageConv (Individual) | T5 embedding | 0.1 | Yes | Yes | 0.24 | 1.33 | 0.49 | 2.27 | 0.21 | 1.35 | 0.62 | 0.88 |
| 12 | SageConv | T5 embedding | 0.1 | Yes | No | 0.75 | 1.05 | 0.41 | 1.25 | 0.22 | 1.54 | 0.31 | 1.04 |

Table S11. Ablation study of UniMutStab on single point mutations.

| ID | Model | Features | | | | S462_direct | | S462_inverse | | S72 | | S98 | |
| --- | --- | --- | --- | --- | --- | --- | --- | --- | --- | --- | --- | --- | --- |
|  |  | Node features | Contact Map Cutoff | Edge | Shared  Weight | PCC | RMSE | PCC | RMSE | PCC | RMSE | PCC | RMSE |
| 1 | SageConv （UniMutStab） | T5 embedding | 0.1 | Yes | Yes | 0.41 | 1.73 | 0.40 | 1.69 | 0.66 | 2.45 | 0.46 | 1.25 |
| 2 | SageConv | Onehot | 0.1 | Yes | Yes | 0.20 | 1.85 | 0.16 | 2.02 | 0.14 | 2.66 | 0.01 | 1.40 |
| 3 | SageConv | T5 embedding | 0.1 | No | Yes | 0.36 | 1.71 | 0.36 | 1.72 | 0.59 | 2.42 | 0.41 | 1.39 |
| 4 | SageConv | T5 embedding | 0.9 | Yes | Yes | 0.39 | 1.77 | 0.38 | 1.69 | 0.58 | 2.48 | 0.43 | 1.26 |
| 5 | SageConv | T5 embedding | 0.5 | Yes | Yes | 0.38 | 1.75 | 0.37 | 1.70 | 0.69 | 2.45 | 0.38 | 1.33 |
| 6 | SageConv | T5 embedding | 0.05 | Yes | Yes | 0.38 | 1.71 | 0.38 | 1.73 | 0.54 | 2.51 | 0.51 | 1.20 |
| 7 | GATConv | T5 embedding | 0.1 | Yes | Yes | 0.37 | 1.76 | 0.34 | 1.74 | 0.66 | 2.45 | 0.46 | 1.25 |
| 8 | GraphConv | T5 embedding | 0.1 | Yes | Yes | 0.30 | 1.76 | 0.34 | 1.79 | 0.57 | 2.46 | 0.39 | 1.30 |
| 9 | ChebConv | T5 embedding | 0.1 | Yes | Yes | 0.37 | 1.75 | 0.38 | 1.71 | 0.54 | 2.44 | 0.52 | 1.19 |
| 10 | GINConv | T5 embedding | 0.1 | Yes | Yes | 0.38 | 1.72 | 0.39 | 1.71 | 0.53 | 2.54 | 0.62 | 1.12 |
| 11 | SageConv (Individual) | T5 embedding | 0.1 | Yes | Yes | 0.35 | 1.78 | 0.35 | 1.74 | 0.74 | 2.55 | 0.37 | 1.30 |
| 12 | SageConv | T5 embedding | 0.1 | Yes | No | 0.21 | 1.92 | 0.18 | 1.79 | 0.54 | 2.63 | 0.33 | 1.50 |

Table S12. UniMutStab Runingtime test.

| Protein length | 100 | 500 | 1000 |
| --- | --- | --- | --- |
| UniMutStab | 2.36s | 4.33s | 11.51s |

The average of 100 test results.


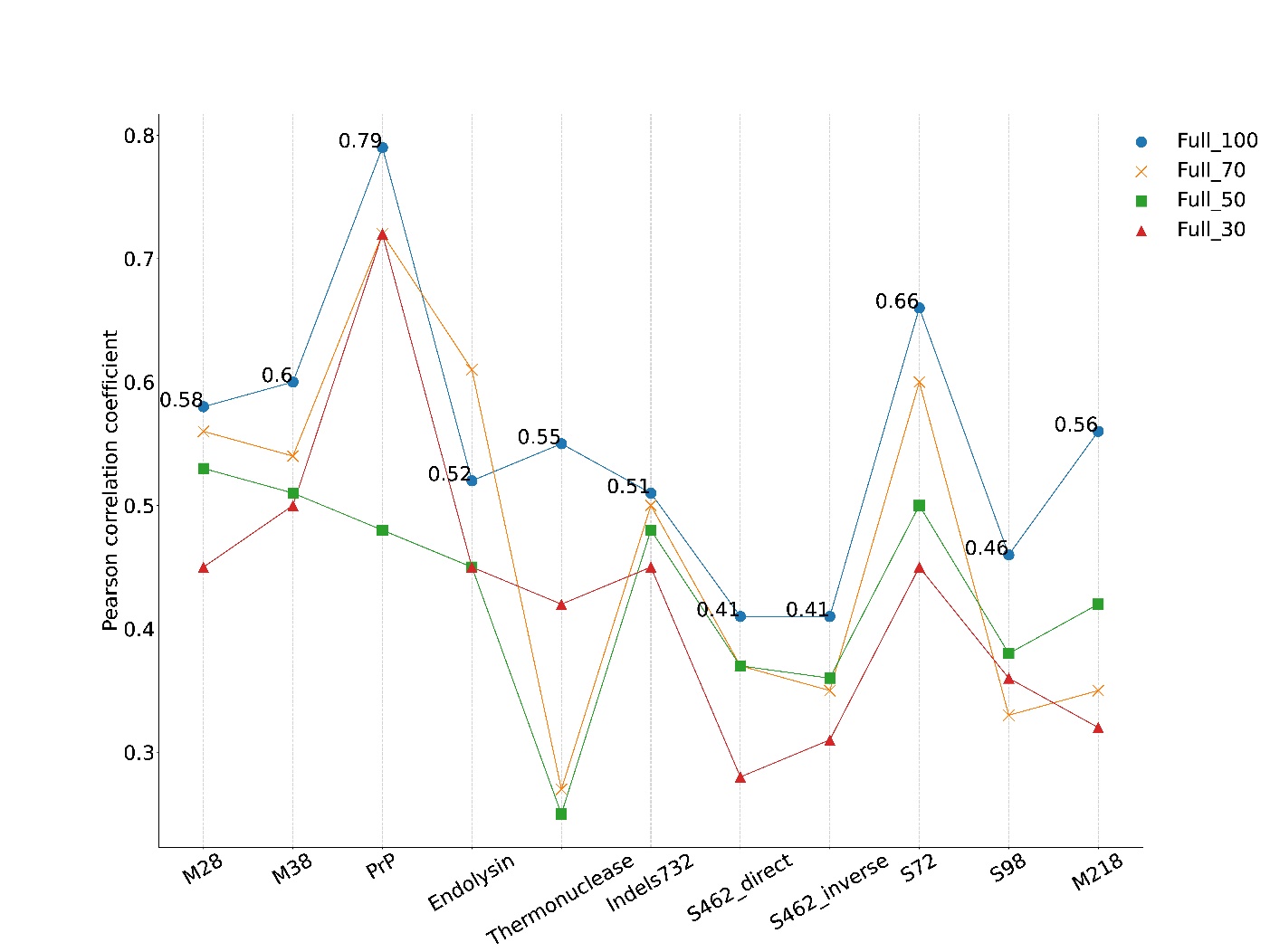


Figure S2. Performance of the UniMutStab framework on various datasets trained on 100%, 70%, ，50% and 30% of the training set.

Pseudocode of UniMutStab

# using prot_t5 model to get amino acids embedding as node feature

function read_node_feature(sequence)

node_feature = Prot_t5_EmbeddingExtraction(sequence)

return node_feature

end function

# using SPOT-Contact-LM model to get Amino Acid Residue Contact Probability Map

function read_contact_map(sequence)

contact_prob_map = SPOT_Contact_LM_predict(sequence)

return contact_prob_map

end function

# generate_graph

function graph_generation(contact_prob_map, cutoff, node_feature)

adjacency_matrix = write_adjacency_matrix(contact_prob_map, cutoff)

edge_feature_matrix = write_edge_feature_matrix(contact_prob_map)

graph = write_graph(adjacency_matrix, node_feature, edge_feature_matrix)

return graph

end function

# ProteinRegressionModel

function ProteinRegressionModel(n_conv, m_fcn, graph_wild, graph_mutated)

for i = 1 to n_conv:

node_feature_wild = Convolution(graph_wild, graph_wild.node_feature, graph_wild.edge_feature)

node_feature_wild = LayerNorm(node_feature_wild)

node_feature_wild = Activation(node_feature_wild)

node_feature_wild = Dropout(node_feature_wild)

end for

for i = 1 to n_conv:

node_feature_mutated = Convolution(graph_mutated, graph_mutated.node_feature, graph_mutated.edge_feature)

node_feature_mutated = LayerNorm(node_feature_mutated)

node_feature_mutated = Activation(node_feature_mutated)

node_feature_mutated = Dropout(node_feature_mutated)

end for

node_feature_wild_mean = Pooling(node_feature_wild)

node_feature_mutated_mean = Pooling(node_feature_mutated)

embedding = Concatenate(node_feature_wild_mean, node_feature_mutated_mean)

for i = 1 to m_fcn:

embedding = Linear(embedding)

embedding = LayerNorm(embedding)

embedding = Activation(embedding)

embedding = Dropout(embedding)

end for

ddg = Regression_linear(embedding)

return ddg

end function
